# Supplementary figures and images for: Identification and Characterization of Three Novel Iflaviruses in the Cabbage Whitefly Aleyrodes proletella
Source: Insects. 2025 Mar 22;16(4):335. doi: 10.3390/insects16040335 (PMC12027991; doi:10.3390/insects16040335)

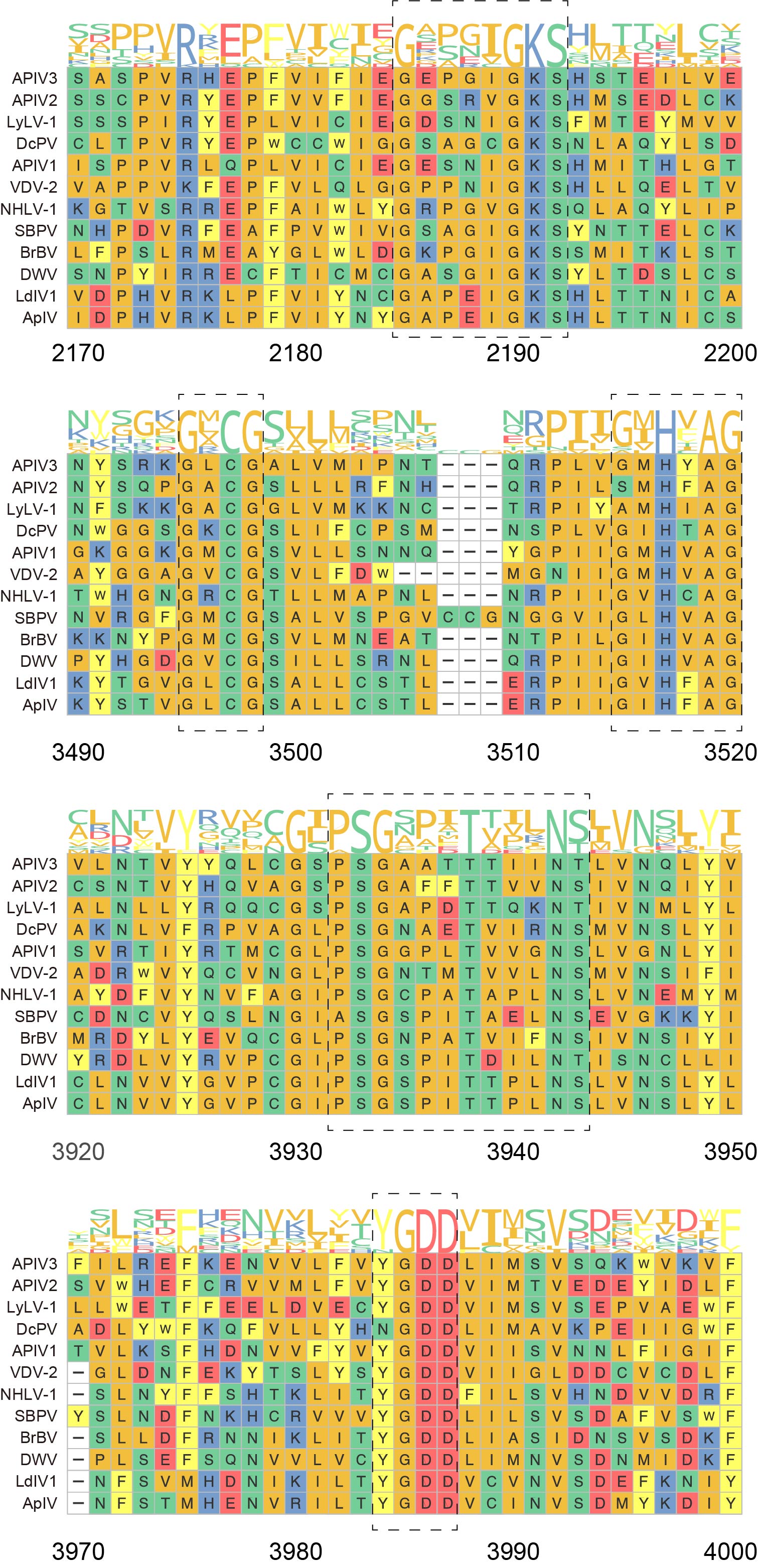

Supplement: Supplementary file 1 [file insects-16-00335-s001.zip › Supplementary Figure S1.jpg]

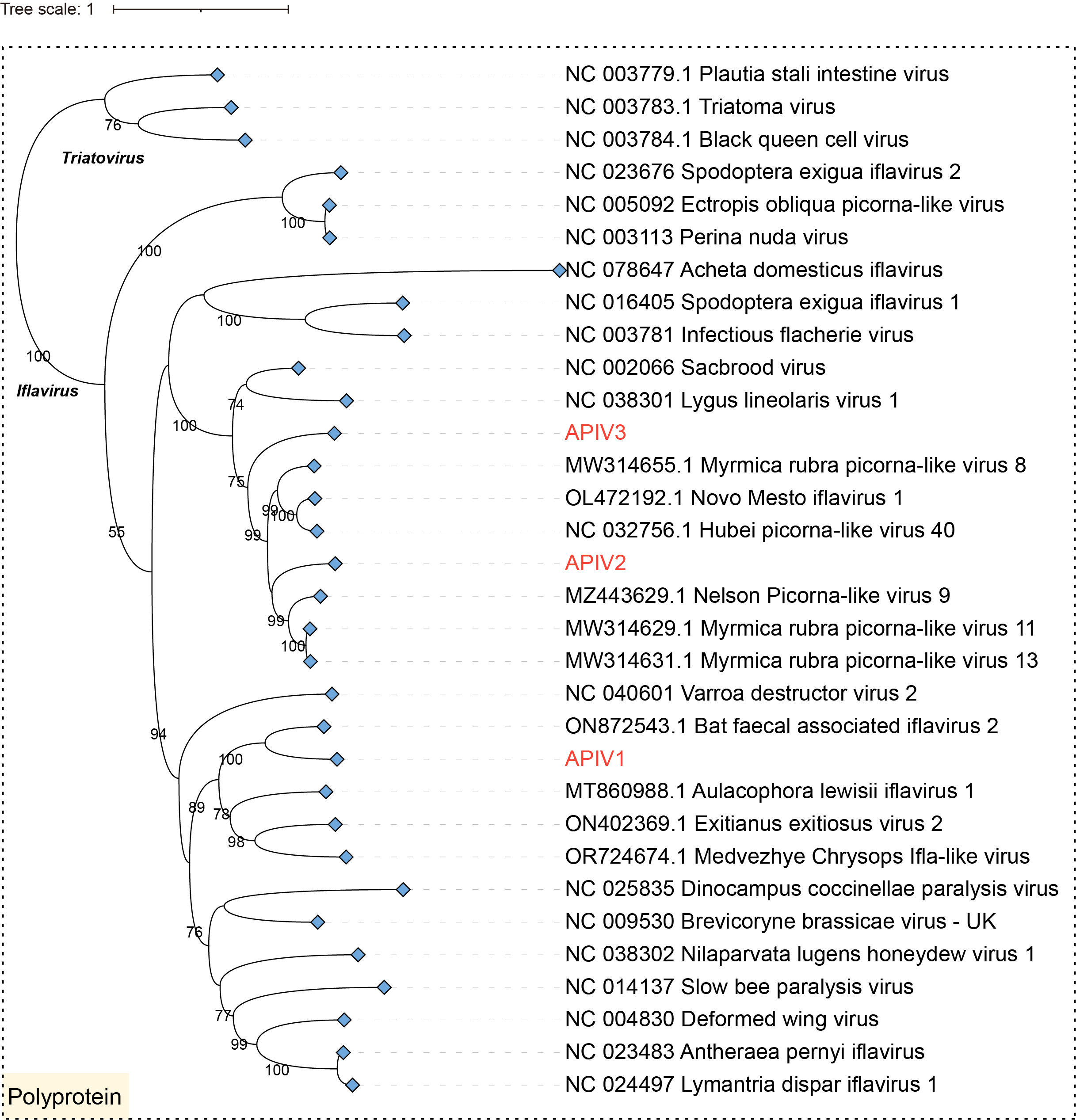

Supplement: Supplementary file 1 [file insects-16-00335-s001.zip › Supplementary Figure S2.jpg]
